# Supplementary figures and images for: Environmental DNA Metabarcoding in Marine Ecosystems: Global Advances, Methodological Challenges, and Applications in the MENA Region
Source: Biology (Basel). 2025 Oct 22;14(11):1467. doi: 10.3390/biology14111467 (PMC12649856; doi:10.3390/biology14111467)

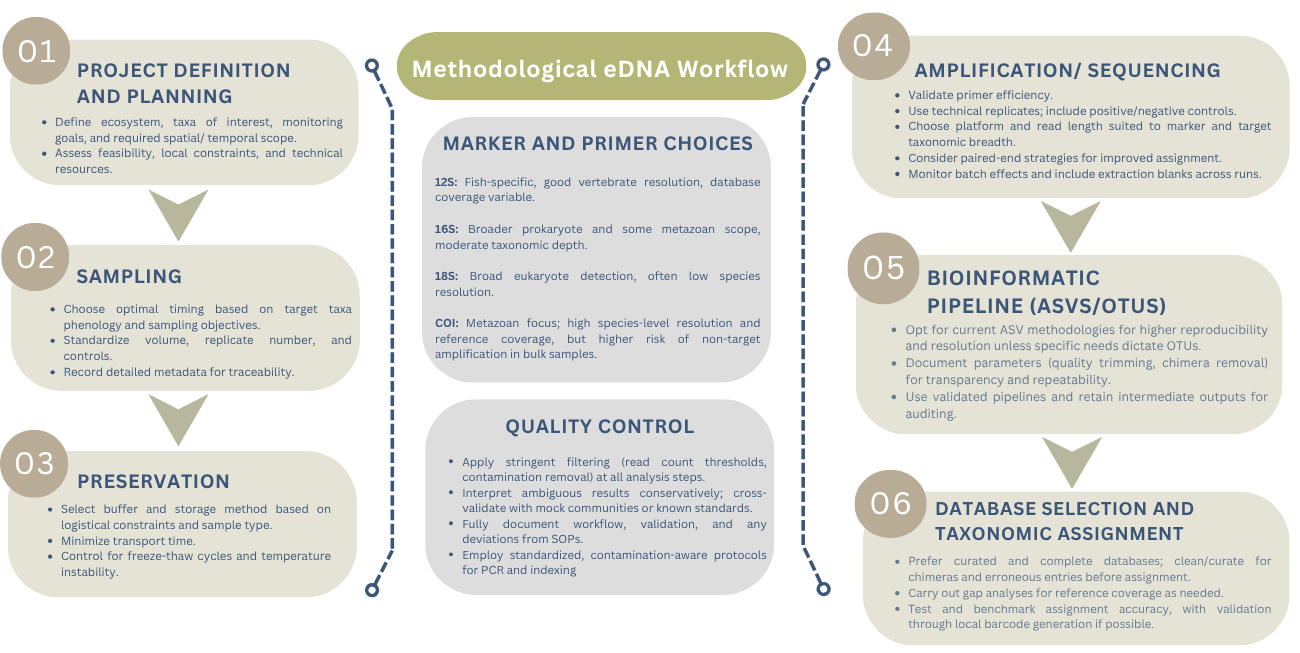

Supplement: Supplementary file 1 [file biology-14-01467-s001.zip › biology-3889297-supplementary.png]
